# Dissection of Malonyl-Coenzyme A Reductase of *Chloroflexus aurantiacus* Results in Enzyme Activity Improvement

**DOI:** 10.1371/journal.pone.0075554

**Published:** 2013-09-20

**Authors:** Changshui Liu, Qi Wang, Mo Xian, Yamei Ding, Guang Zhao

**Affiliations:** 1 Qingdao Institute of Bioenergy and Bioprocess Technology, Chinese Academy of Sciences, Qingdao, Shandong, China; 2 Key Laboratory of Biobased Materials, Chinese Academy of Sciences, Qingdao, Shandong, China; 3 University of Chinese Academy of Sciences, Beijing, China; 4 Institute of Oceanology, Chinese Academy of Sciences, Qingdao, Shandong, China; University of Edinburgh, United Kingdom

## Abstract

The formation of fusion protein in biosynthetic pathways usually improves metabolic efficiency either channeling intermediates and/or colocalizing enzymes. In the metabolic engineering of biochemical pathways, generating unnatural protein fusions between sequential biosynthetic enzymes is a useful method to increase system efficiency and product yield. Here, we reported a special case. The malonyl-CoA reductase (MCR) of *Chloroflexus aurantiacus* catalyzes the conversion of malonyl-CoA to 3-hydroxypropionate (3HP), and is a key enzyme in microbial production of 3HP, an important platform chemical. Functional domain analysis revealed that the N-terminal region of MCR (MCR-N; amino acids 1-549) and the C-terminal region of MCR (MCR-C; amino acids 550-1219) were functionally distinct. The malonyl-CoA was reduced into free intermediate malonate semialdehyde with NADPH by MCR-C fragment, and further reduced to 3HP by MCR-N fragment. In this process, the initial reduction of malonyl-CoA was rate limiting. Site-directed mutagenesis demonstrated that the TGXXXG(A)X(1-2)G and YXXXK motifs were important for enzyme activities of both MCR-N and MCR-C fragments. Moreover, the enzyme activity increased when MCR was separated into two individual fragments. Kinetic analysis showed that MCR-C fragment had higher affinity for malonyl-CoA and 4-time higher *K*_cat_/*K*_*m*_ value than MCR. Dissecting MCR into MCR-N and MCR-C fragments also had a positive effect on the 3HP production in a recombinant *Escherichia coli* strain. Our study showed the feasibility of protein dissection as a new strategy in biosynthetic systems.

## Introduction

**Figure 1:**
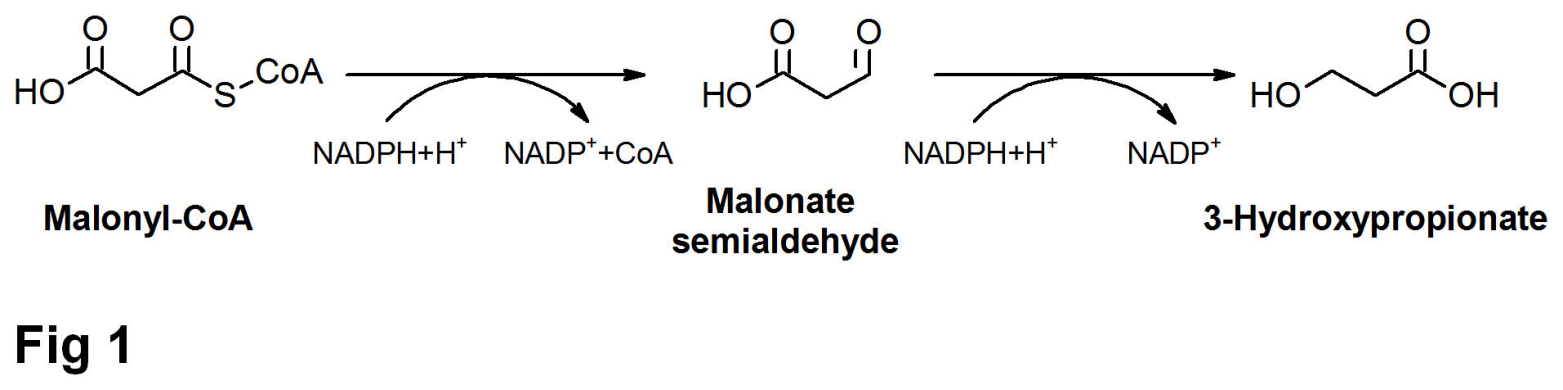
The reaction catalyzed by MCR.

The malonyl-coenzyme A (CoA) reductase (MCR) of phototrophic green nonsulfur bacterium *Chloroflexus aurantiacus* is a bifunctional enzyme with alcohol dehydrogenase and aldehyde dehydrogenase (CoA-acylating) activities. MCR catalyzes the two-step reduction of malonyl-CoA with NADPH to 3-hydroxypropionate (3HP), and malonate semialdehyde was suggested as the likely free intermediate (Fig. 1) [1]. 3HP, the reaction product of MCR, is an important platform chemical ranked in the list of top 12 value added chemicals from biomass released by the US Department of Energy [2]. MCR has been used in engineered strains producing 3HP or poly(3HP), a biocompatible and biodegradable thermoplastic. However, the low enzyme activity of MCR expressed in recombinant strains limited the 3HP and poly(3HP) production [3,4].

In biosynthetic pathways, the formation of fusion proteins catalyzing sequential reactions usually improves metabolic efficiency either by channeling intermediates or by localizing active sites in close proximity [5]. Linking genes together to generate a functional fusion protein has become an attractive strategy for metabolic engineering purposes, and was successfully applied in microbial production of resveratrol [6], miltiradiene [7], etc.

Here, we report a special case that MCR, a natural fusion protein of two short-chain dehydrogenase/reductase, has higher enzyme activity when separated into two fragments. The *in vivo* dissection of MCR also resulted in improved 3HP production by a recombinant *Escherichia coli* strain.

## Materials and Methods

### Plasmid construction

All strains and plasmids used in this study were listed in Table 1. All PCR were done using PrimeSTAR HS DNA Polymerase (TAKARA, Dalian, China). The *mcr* gene of *C. aurantiacus* J-10-fl (NCBI accession No. YP_001636209) was synthesized and inserted into the *Bam*HI and *Xho*I sites of pETDuet-1. The plasmid pMCR-N was constructed by cloning the *mcr-N* into *Bam*HI and *Hin*dIII sites of pETDuet-1 with primers 233 (5’-CATGGATCCGAGCGGAACAGGACGAC-3’) and 253 (5’-CTGAAGCTTATCCGACCGATGCACTGC-3’). The plasmid pMCR_1-485_ was constructed by cloning the *mcr*_1-485_ into *Bam*HI and *Hin*dIII sites of pETDuet-1 with primers 233 and 289 (5’- CAGTAAGCTTAGTGACGCCACACACGAATG-3’). The plasmid pMCR-C and pMCR-N-C were constructed by cloning the *mcr-C* into *Bgl*II and *Xho*I sites of pETDeut-1 and pMCR-N, respectively, with primers 295 (5’-CATCAGATCTCCATCACCATCATCACCATCACAGCGCCACCACCGGCGCA-3’) and 126 (5’-CCCTCGAGGAATTTACACGGTAATCGC-3’). The plasmid pMCR_550-1170_ was constructed by cloning the *mcr*_550-1170_ into *Bgl*II and *Xho*I sites of pETDeut-1 with primers 295 and 292 (5’-CCCTCGAGTTACGCACGCCGGGTCAGATC-3’). The following point mutations in the putative NADPH binding sites and active sites were introduced into pMCR-N, pMCR-C, or pMCR: G4R, G16R, Y171G/K175G, G554R, G579R, and Y737G/K741G. The pMCR-G4R was constructed by cloning *mcr-N* into *Bam*HI and *Hin*dIII sites of pETDuet-1 with primers 326 (5’-CATGGATCCGAGCGGAACAAGACGACTGGCAGGAAAG-3’) and 253. All other mutagenesis were performed with pMCR-N, pMCR-C, or pMCR as a template using primers 327 (5’-GAAAGATTGCGTTAATTACCAGAGGCGCCGGCAATATCGGCAG-3’) and 328 (5’-GGTAATTAACGCAATCTTTCC-3’) for G16R, 234 (5’-GCAGCACCAGGGGTGACACCCGGAATCCGCCCGTAGTAC-3’) and 235 (5’-GGTGTCACCCCTGGTGCTGCTCTTAATGCTCTATC-3’) for Y171G/K175G, 329 (5’-CATCACAGCGCCACCACCAGAGCACGCAGTGCATCGGTCG-3’) and 330 (5’-GGTGGTGGCGCTGTGATG-3’) for G554R, 331 (5’-GGAAAGTTGCCTTGATTACCAGAGGCAGCGCCGGTATTGGTG-3’) and 332 (5’-GGTAATCAAGGCAACTTTCC-3’) for G579R, 237 (5-CCAGCACCCGAGACGGCACCATCGGCACGGTTGGGGTAG-3’) and 238 (5’-GGTGCCGTCTCGGGTGCTGGTCAGCGGGCAATG-3’) for Y737G/K741G. The QuikChange Site-Directed Mutagenesis Kit (Stratagene) was used according to the manufacturer’s protocol.

**Table 1:** Bacteria strains and plasmids used in this study.

**Strains or plasmid**	**Description**	**Source**
**Strains**		
*E. coli* DH5α	F^-^ supE44 Δ*lacU*169 (*ϕ*80 *lacZ* Δ*M15*) hsdR17 recA1 *endA1* gyrA96 *thi*-1 relA1, host for the preparation of plasmid DNA	Invitrogen
*E. coli* BL21(DE3)	F^-^ *ompT gal dcm lon hsdSB* (rB^-^ mB^-^) λ(DE3), host for protein expression and 3HP production	Invitrogen
**Plasmids**		
pETDuet-1	rep_pBR322_ Amp^R^ *lacI* P_T7_	Novagen
pA-*accADBC*	rep_p15A_ Cm^R^ *lacI* P_T7_ *accA* P_T7_ *accD* P_T7_ *accBC*	[10]
pMCR	rep_pBR322_ Amp^R^ *lacI* P_T7_ *His*_*6*_ *-mcr*	this study
pMCR-N	rep_pBR322_ Amp^R^ *lacI* P_T7_ *His*_*6*_ *-mcr*_1-549_	this study
pMCR_1-485_	rep_pBR322_ Amp^R^ *lacI* P_T7_ *His*_*6*_ *-mcr*_1-485_	this study
pMCR-C	rep_pBR322_ Amp^R^ *lacI* P_T7_ *His*_*6*_ *-mcr*_550-1219_	this study
pMCR_550-1170_	rep_pBR322_ Amp^R^ *lacI* P_T7_ *His*_*6*_ *-mcr*_550-1170_	this study
pMCR-N-C	rep_pBR322_ Amp^R^ *lacI* P_T7_ *His*_*6*_ *-mcr*_1-549_ P_T7_ *His*_*6*_ *-mcr*_550-1219_	this study
pMCR-C-554	rep_pBR322_ Amp^R^ *lacI* P_T7_ *His*_*6*_ *-mcr*_550-1219_ G554R	this study
pMCR-C-579	rep_pBR322_ Amp^R^ *lacI* P_T7_ *His*_*6*_ *-mcr*_550-1219_ G579R	this study
pMCR-C-737/741	rep_pBR322_ Amp^R^ *lacI* P_T7_ *His*_*6*_ *-mcr*_550-1219_ Y737G K741G	this study
pMCR-N-4	rep_pBR322_ Amp^R^ *lacI* P_T7_ *His*_*6*_ *-mcr*_1-549_ G4R	this study
pMCR-N-16	rep_pBR322_ Amp^R^ *lacI* P_T7_ *His*_*6*_ *-mcr*_1-549_ G16R	this study
pMCR-N-171/175	rep_pBR322_ Amp^R^ *lacI* P_T7_ *His*_*6*_ *-mcr*_1-549_ Y171G K175G	this study
pMCR-737/741	rep_pBR322_ Amp^R^ *lacI* P_T7_ *His*_*6*_ *-mcr* Y737G K741G	this study
pMCR-554	rep_pBR322_ Amp^R^ *lacI* P_T7_ *His*_*6*_ *-mcr* G554R	this study
pMCR-579	rep_pBR322_ Amp^R^ *lacI* P_T7_ *His*_*6*_ *-mcr* G579R	this study
pMCR-171/175	rep_pBR322_ Amp^R^ *lacI* P_T7_ *His*_*6*_ *-mcr* Y171G K175G	this study
pMCR-4	rep_pBR322_ Amp^R^ *lacI* P_T7_ *His*_*6*_ *-mcr* G4R	this study
pMCR-16	rep_pBR322_ Amp^R^ *lacI* P_T7_ *His*_*6*_ *-mcr* G16R	this study

### Western blot

The *E. coli* BL21(DE3) strains carrying expression plasmids for MCR and its derivative proteins were grown at 37°C in minimal medium, which contains 30 mM potassium phosphate buffer (pH 7.0), 2 g/L (NH_4_)_2_SO_4_, 0.2 g/L MgSO_4_·7H_2_O, 0.5 g/L yeast extract, and 20 g/L glucose. The cells were induced at ~0.6 OD_600_ with 0.05 mM IPTG and further cultured for 4 h. Then cells were collected by centrifugation, washed twice with PBS buffer and resuspended in the same buffer. The cells were disrupted by sonication, the cell lysates were centrifuged, and the supernatants were used for immunoblot analysis. The concentrations of soluble protein samples were determined using the Bradford Protein Assay Kit (Tiangen, China) using bovine serum albumin as a standard. 20 µg of soluble proteins were applied onto SDS-PAGE and analyzed by western blot using anti-His_6_ antibody.

### Protein purification

The His_6_-tagged MCR wild-type and derivative proteins were purified from *E. coli* BL21(DE3) cell extracts using Ni-NTA His·Bind Column (Novagen) according to the manufacturer’s instruction. The eluted protein was dialyzed in PBS buffer overnight. Protein concentrations were determined as above. The purified proteins were analyzed by SDS-PAGE and visualized by Coomassie Blue staining.

### Mass spectrometry analysis

The enzyme reaction mixture (300 µl) contained 100 mM Tris-HCl (pH 7.8), 2 mM MgCl_2_, 0.4 mM NADPH, and 0.05 nmol (each) of purified protein(s). The reaction was started by the addition of 0.15 mM malonyl-CoA and was incubated at 57°C for 2 min. Then the reaction was stopped by addition of 1.2 mL acetonitrile. After centrifugation, the supernatant was analyzed by high performance liquid chromatography-triple quadrupole mass spectrometer (HPLC-QQQMS). Mass spectra were acquired on an Agilent 1290/6430 instrument (Agilent, CA, USA) in the negative-ion mode. Full scan mass spectra were obtained, and the ion chromatograms of malonyl-CoA, malonate semialdehyde, and 3HP were extracted using the m/z of 862, 87, and 89, respectively.

### Enzyme assays

The assay mixture (300 µL) contained 100 mM Tris-HCl (pH7.8), 2 mM MgCl_2_, 0.4 mM NADPH, and 0.05 nmol (each) of purified protein(s). The reaction was started by the addition of 0.15 mM malonyl-CoA and was incubated at 57°C for 30 s. NADPH concentration was monitored at 365 nm (*ε*_365nm_=3.4x 10^3^ M^-1^cm^-1^) using Varian, Cary 50 Bio UV-Visible Spectrophotometer, and the enzyme activity was calculated according to the speed of NADPH oxidation.

To determine the optimal temperature for His_6_-tagged MCR and MCR-C, the reactions were incubated at a series of temperatures (32, 37, 42, 47, 52, 57, 62, and 67°C). To determine the optimal pH, Tris-HCl buffers with different pH (pH 6.6, 7.2, 7.8, 8.4, and 9.0) were tested. To determine the *K*_*m*_ and *K*_cat_ values, a series of malonyl-CoA concentrations (0.05, 0.058, 0.067, 0.083, 0.1, 0.125, and 0.15 mM) were used and the reactions were carried out at 57°C at pH7.8 and pH7.2 for MCR and MCR-C protein, respectively.

### 3HP production

The strains *E. coli* BL21(DE3)/pA-*accADBC*/pMCR and *E. coli* BL21(DE3)/pA-*accADBC*/pMCR-N-C were grown overnight in LB broth and 1:100 diluted into 500 ml baffled Erlenmeyer flasks containing 100 mL of minimal medium as described above. The recombinant cells were induced at ~0.6 OD_600_ with 0.05 mM IPTG. IPTG (0.05 mM), biotin (40 mg/L), and NaHCO_3_ (20 mM) were added periodically every 12 h until 48 h. The western blot was performed as above. The enzyme activity was determined using total soluble proteins at pH7.2 at 37 °C. The 3HP concentration in medium was determined by HPLC as described previously [3]. All shake flask experiments were carried out in triplicates.

## Results and Discussion

### Sequence analysis of MCR

MCR is composed of 1219 amino acid residues, and does not share significant sequence similarity with any known protein [1]. Conserved domains analysis revealed two short-chain dehydrogenase/reductase (SDR) domains, composed of amino acids 1-283 and amino acids 550-782. In each SDR domain, there are two sequence motifs (TGXXXG(A)X(1-2)G) forming Rossmann fold structure for NAD(P) (H) binding [8], and a conserved YXXXK motif, implicated as the catalytic site in SDR family proteins [9]. Taken into account that MCR protein catalyzes a two-step reaction of malonyl-CoA reduction to 3HP, it is speculated that the N-terminal and C-terminal regions of MCR protein are functionally distinct, responsible for the alcohol dehydrogenase and aldehyde dehydrogenase (CoA-acylating) activity, respectively. Thus the N-terminal region of MCR (amino acids 1-549) was defined as MCR-N and the C-terminal region (amino acids 550-1219) as MCR-C.

### Analysis of functional domains in the MCR protein

**Figure 2:**
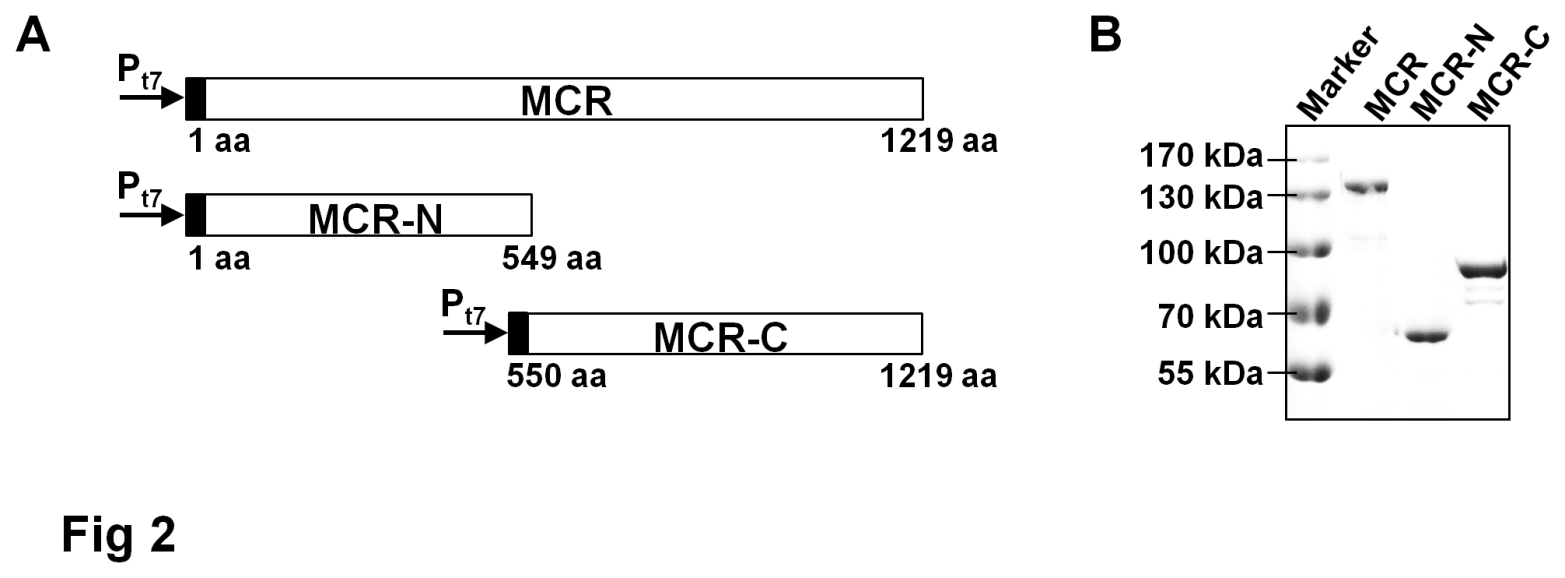
Expression of the recombinant MCR protein and its derivatives. (A) Schematic representation of MCR, MCR-N, and MCR-C proteins fused to a His_6_-tag (black). (B) Western blot analysis of MCR, MCR-N, and MCR-C recombinant proteins expressed in *E. coli* BL21(DE3) strain. The soluble protein samples were applied onto SDS-PAGE and detected using anti-His_6_ antibody.

To elucidate the function of each domain of MCR, an appropriate set of expression vectors was constructed for His_6_-tagged *MCR*, *MCR-N*, and *MCR-C* genes under the IPTG-inducible T7 promoter (Figure 2A and Table 1). The proteins expression in *E. coli* BL21(DE3) was confirmed by immunoblot analysis of total soluble proteins using anti-His_6_ antibody. These proteins were observed as 135-kDa, 62-kDa, and 83-kDa bands, respectively (Figure 2B).

**Figure 3:**
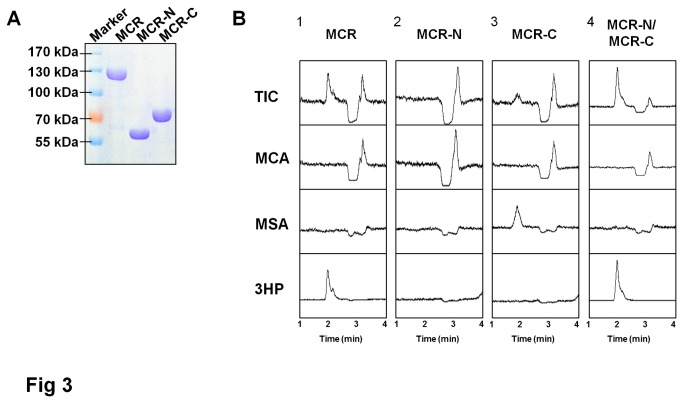
Functional domain analysis of MCR protein. (A) SDS-PAGE of purified MCR, MCR-N, and MCR-C proteins. (B) *In* *vitro* analysis of malonyl-CoA reductase activity. Enzyme activities were assayed as described in Materials and Methods. The reaction products of MCR, MCR-N, MCR-C, and MCR-N/MCR-C were analyzed using LC-MS. Representative results from duplicated independent experiments are shown, TIC, total ion chromatogram. MCA, malonyl-CoA. MSA, malonate semialdehyde.

The His_6_-tagged MCR, MCR-N, and MCR-C proteins were purified using Ni-NTA His·Bind Column and analyzed by SDS-PAGE (Figure 3A). The enzymatic activities were examined *in vitro* using malonyl-CoA as a substrate and NADPH as co-factor at 57°C. NADPH oxidation was monitored at 365 nm (*ε*_365nm_=3.4x 10^3^ M^-1^cm^-1^), and the product was analyzed by LC-MS. The MCR-N fragment showed no conversion of malonyl-CoA (Figure 3B, panel 2). In contrast, NADPH was oxidated when MCR-C was used, and LC-MS analysis showed a new peak with a mass of 87 Da (Figure 3B, panel 3), corresponding to the mass of ionized malonate semialdehyde, demonstrating that the MCR-C protein can catalyze the reduction of malonyl-CoA to malonate semialdehyde solely. Since malonate semialdehyde is not commercially available, the activity of MCR-N fragment was tested using malonyl-CoA as substrate along with MCR-C and the production of 3HP should indicate the activity of MCR-N fragment. As shown in Figure 3B panel 4, malonate semialdehyde was transformed into 3HP. Taken together, these results demonstrated that MCR-N and MCR-C are functionally distinct, representing alcohol dehydrogenase and aldehyde dehydrogenase (CoA-acylating) activity, respectively. Furthermore, the malonate semialdehyde was experimentally identified as free intermediate in MCR reaction.

The classical SDR has a chain length of about 250 amino acid residues, which is much shorter than MCR-N and MCR-C fragments. Truncated derivatives of MCR-N and MCR-C proteins, like MCR_1-485_ and MCR_550-1170_, were also tested. The deletions of C-terminal amino acid residues in both MCR-C and MCR-N fragments completely eliminated the corresponding enzyme activity, suggesting that the C-terminal sequences in both fragments have some unknown but important role, such as stabilizing the protein structure, binding with the substrates, etc.

### Kinetic analyses of the MCR-C protein fragment

**Figure 4:**
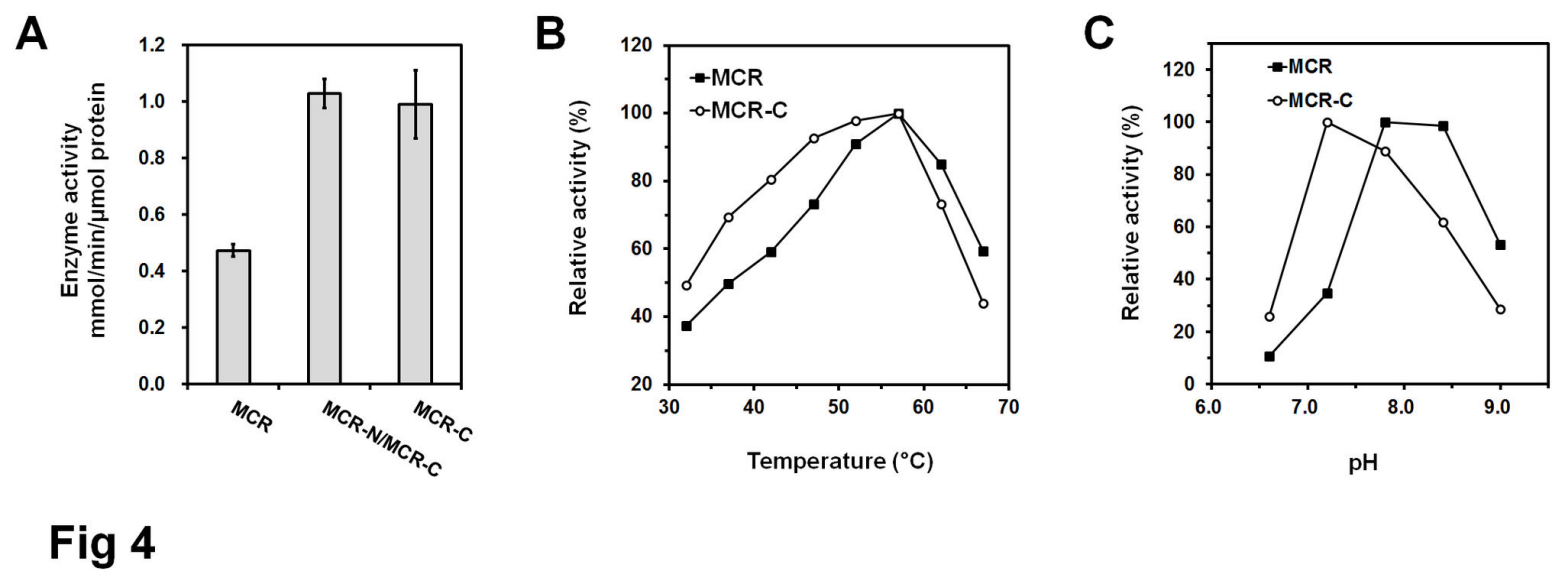
Kinetic analysis of MCR and MCR-C proteins. (A) Enzyme activity comparison of MCR, MCR-N/MCR-C, and MCR-C. 0.05 nmol (each) of MCR, MCR-N/MCR-C, and MCR-C were used to catalyze the reactions at pH7.8 at 57°C, respectively. Date represent mean ± standard deviation (N=3). (B) Effects of temperature on enzymatic activity of His_6_-tagged MCR and MCR-C proteins. The reactions were performed at the condition of pH 7.8 (Tris-HCl). For MCR, 100% corresponds to the enzyme activity of 0.47 mmol/min/μmol protein at 57°C; for MCR-C, 100% corresponds to the enzyme activity of 0.99 mmol/min/μmol protein at 57°C. (C) Effects of pH on enzymatic activity of His_6_-tagged MCR and MCR-C proteins. The reactions were performed at 57°C. For MCR, 100% corresponds to the enzyme activity of 0.47 mmol/min/μmol protein at the condition of pH7.8 (Tris-HCl); for MCR-C, 100% corresponds to the enzyme activity of 1.12 mmol/min/μmol protein at the condition of pH7.2 (Tris-HCl).

In Figure 3B, it was observed that the peak of 3HP product was higher and the peak of malonyl-CoA substrate was smaller in panel 4 when compared with panel 1. This phenomenon indicated that the enzymatic activity increased somehow when MCR was separated into two proteins. Though the mass spectrometric response is quantitative, this data was unable to be used in a quantitative manner because of the unavailability of malonate semialdehyde standard. So the kinetic analyses were carried out *in vitro* spectrophotometrically. To test this speculation, enzyme activities of MCR and MCR-N/MCR-C were determined using 0.05 nmol (each) of purified protein(s) at pH7.8 at 57°C. As shown in Figure 4A, the MCR protein and MCR-N/MCR-C mixture catalyzed the reduction of malonyl-CoA with activities of 0.47 ± 0.05 and 1.02 ± 0.05 mmol/min/μmol protein, respectively (*p*<0.01), suggesting that the combined activity of MCR-N and MCR-C fragments is greater than that of wild type MCR protein.

In reactions catalyzed by either MCR or MCR-N/MCR-C, malonate semialdehyde was not accumulated (Figure 3B, panel 1 and 4), and the kinetics of malonyl-CoA dependent oxidation of NADPH followed normal Michaelis-Menten kinetics. No clear sign of a biphasic reaction was observed, suggesting that the reduction of the malonate semialdehyde to 3HP is fast and the reduction of malonyl-CoA to semialdehyde is rate-limiting step. So we focused on the MCR-C fragment and determined its activity *in vitro*. Malonyl-CoA was reduced to malonate semialdehyde at pH7.8 at 57°C by MCR-C fragment with an activity of 0.99 ± 0.12 mmol/min/μmol protein, which is more than 2-times higher than that of MCR protein (Figure 4A).

The optimal temperature and pH for MCR-C were determined. The enzyme activity was highest at 57 °C, and was fairly stable over the temperature range of 37-62 °C (Figure 4B). The optimal pH for activity was pH7.2, showed a significant shift compared with MCR (Figure 4C). MCR-C fragment has an apparent *K*_*m*_ value of 23.8 ± 1.9 µmol for malonyl-CoA, lower than that of MCR (41.7±2.2 µmol), and the *Kcat*/*K*_*m*_ value of MCR-C fragment was about 4-fold higher than MCR protein (Table 2), suggesting that MCR-C fragment has higher affinity for malonyl-CoA and higher catalyzing efficiency than MCR protein. Due to the absence of sequence similarity with other proteins and lack of MCR protein structure information, the mechanistic basis of activity enhancement remains unknown. It is assumed that the N-terminal structure blocks the entrance of C-terminal domain active site to a certain extent in MCR protein, and malonyl-CoA can get into the catalytic center of C-terminal domain more easily when two domains are apart.

**Table 2:** Kinetic analysis of MCR and MCR-C.

	*K*_*m*_ (μM)	*K*_cat_ (s^-1^)	*K*_cat_/*K*_*m*_ (mM^-1^s^-1^)
MCR	41.7±2.2	7.81±0.77	187
MCR-C	23.8±1.9	18.6±2.4	782

The *K*_*m*_ and *K*_cat_ value were determined under the optimal conditions of each enzyme. Date represent mean ± standard deviation (N=3).

### Determination of essential amino acid residues in each fragment

**Figure 5:**
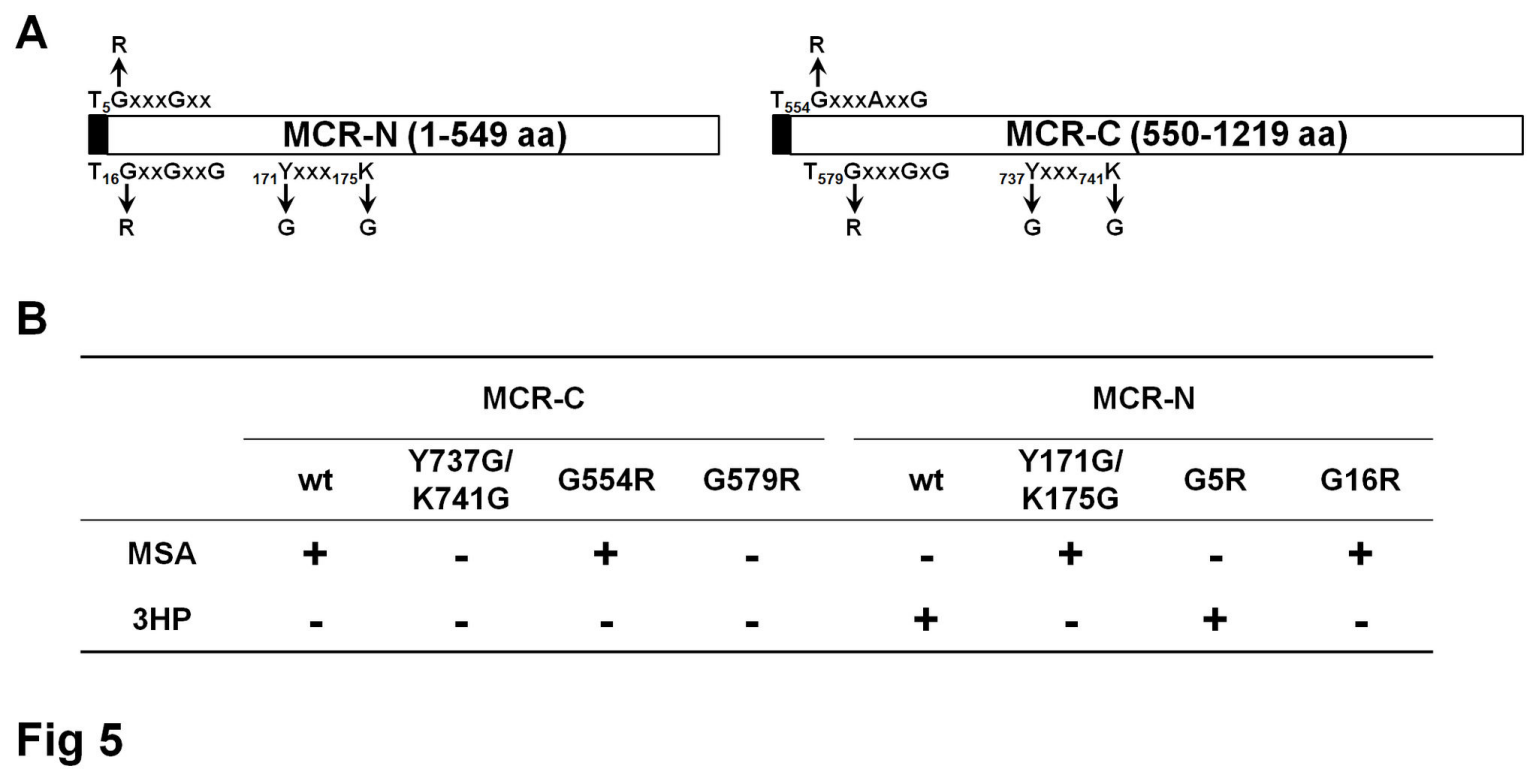
Site-directed mutagenic analysis of the MCR-N and MCR-C proteins. (A) The location of amino acid changed in the MCR-N and MCR-C proteins that were tested. (B) *In* *vitro* analysis of malonyl-CoA reductase activity. Wild-type and mutated proteins were tested and the products were identified by LC-MS. In MCR-N reactions, wild-type MCR-C protein was used to convert malonyl-CoA into malonate semialdehyde, which is the substrate of MCR-N.

Two conserved motifs of NADPH binding site and one of SDR reactive site were found in both MCR-N and MCR-C fragments. To confirm the importance of amino acid residues in these motifs, several point mutation versions of MCR-N or MCR-C fragments were constructed, including G5R, G16R, and Y171G/K175G mutants of MCR-N fragment, G554R, G579R, and Y737G/K741G mutants of MCR-C fragment (Figure 5A). For the MCR-C fragment, G579R and Y737G/K741G completely abolished aldehyde dehydrogenase (CoA-acylating) activity of MCR-C fragment and no malate semialdehyde product could be detected, while the G554R mutant showed the similar activity with MCR-C wild-type fragment (Figure 5B). The activities of MCR-N mutants were tested along with wild-type MCR-C fragment using malonyl-CoA as substrate. The G16R and Y171G/K175G mutants resulted in the MCR-N inactivation as the intermediate malonate semialdehyde but not 3HP was accumulated in the reaction mixture, and the G5R mutant only had a modest effect on reaction velocity and can still converted the malonate semialdehyde into 3HP (Figure 5B). These data suggested that the conserved reactive motif of SDR, YXXXK, is essential for robust alcohol dehydrogenase and aldehyde dehydrogenase (CoA-acylating) activity of the corresponding fragment. Despite the fact that two conserved NADPH binding motifs were found in both MCR-N and MCR-C fragments, only one of them (^15^TGGAGNIG^22^ and^578^TGGSAGIG^585^) has important effects on the reactivity of the corresponding domains. Mutagenesis of reactive motifs and NADPH binding sites in full-length MCR protein also confirmed the above results.

### *In vivo* effect of separated MCR protein

**Figure 6:**
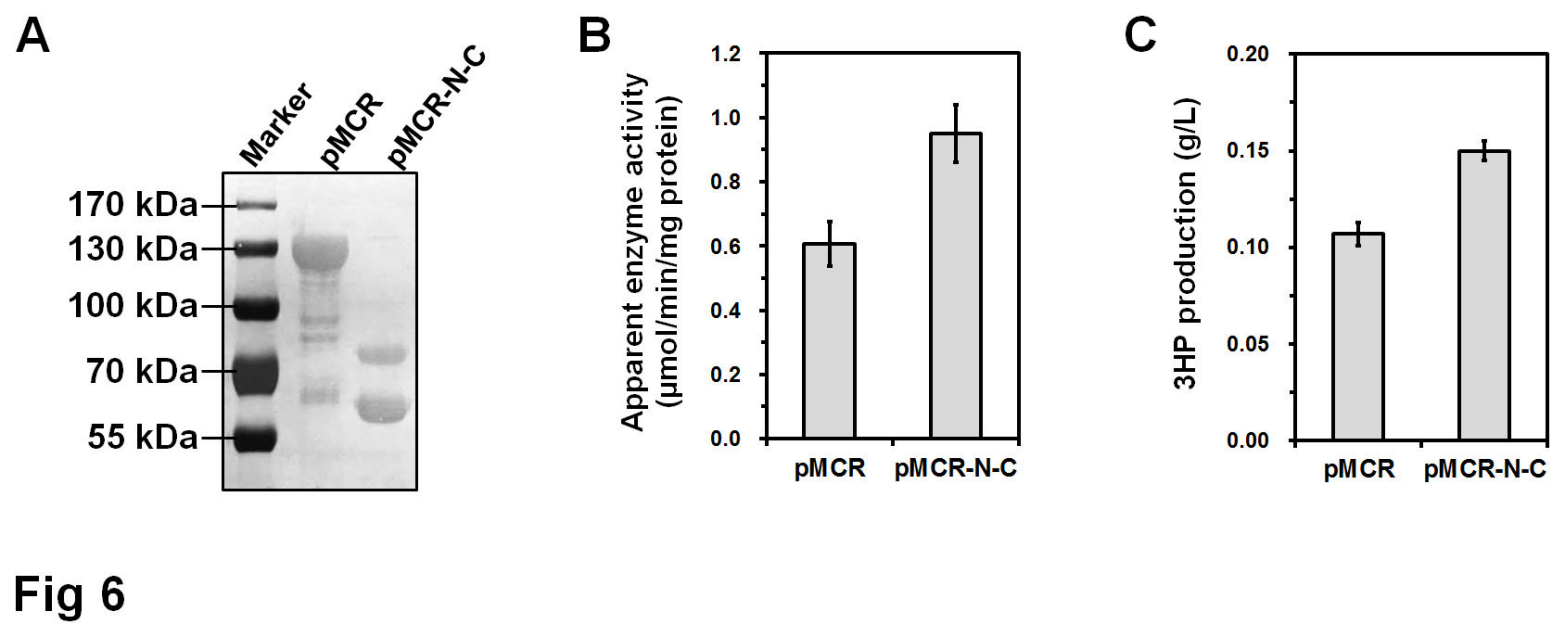
*In vivo* effect of separated MCR protein on protein expression (A), enzyme activity (B), and 3HP production (C). Soluble proteins extracts obtained by sonication of *E. coli* BL21(DE3) cells carrying pMCR or pMCR-N-C were used for western blot analysis and enzyme activity assay. Representative western blot result from triplicated independent experiments is shown. Enzyme activity assay was performed in Tris-HCl buffer pH7.2 at 37°C. Date represent mean ± standard deviation (N=3).

To determine the effect of dissection-dependent MCR activity improvement on 3HP production *in vivo*, two recombinant *E. coli* strains were constructed. One carries the wild-type MCR protein expression vector pMCR, and the other one possesses vector pMCR-N-C to overexpress separated MCR-N and MCR-C fragments. The plasmid pA-*accADBC* carrying acetyl-CoA carboxylase gene [10] was used to channel the carbon into 3HP production in both strains. These strains were grown in minimal medium using glucose as the sole carbon source. After 48 h incubation, the cells were collected and disrupted by sonication, and equal amount of soluble protein extracts were used to determine the protein expression level and enzyme activity. As shown in Figure 6A, the MCR-C fragment was significantly less expressed than full length protein and the comparison of MCR protein with MCR-N fragment showed intensities of the same order of magnitude. The enzyme activity was tested in Tris-HCl buffer pH7.2 at 37 °C to farthest mimic *E. coli* intracellular conditions. The soluble protein extracts from strains carrying pMCR-N-C and pMCR catalyzed the formation of 3HP from malonyl-CoA at apparent activities of 0.95 ± 0.09 and 0.61 ± 0.07 μmol/min/mg protein, respectively (*p*<0.01) (Figure 6B). HPLC analysis of culture supernatant showed that the strain carrying pMCR-N-C accumulated 0.150 ± 0.005 g/L 3HP, while the strain carrying pMCR produced 0.107 ± 0.006 g/L 3HP (*p*<0.01) (Figure 3C). These results demonstrated that the isolated MCR-C fragment, *in vivo* as well as *in vitro*, is more active than the C-terminal domain integrated in the full length MCR protein, and dissection of MCR protein do have a positive effect on microbial 3HP production.

Although the 3HP level is still low, our study showed feasibility to develop a new strategy for metabolic engineering by separating a multifunctional protein into individual fragments. It has some expected advantages. First, the separated fragments may have enhanced overall enzyme activity. For example, some bifunctional oxidoreductases, like AdhE from *Escherichia coli* and Aad/AdhE from *Clostridium acetobutylicum* [11,12], catalyze reactions similar with MCR, the reduction of a CoA thioester of an organic acid to its corresponding alcohol using NADH as a cofactor. Second, expression and folding of large multidomain proteins are typically inefficient in bacteria [13]. The *ols* gene of *Synechococcus* sp. PCC 7002 encodes a large multidomain protein (302 kDa) involved in α-olefin biosynthesis [14]. However, the codon-optimized *ols* gene still cannot be expressed in *E. coli* [15]. A possible solution could be separating this protein into short functional fragments.

## Conclusions

In summary, *Chloroflexus aurantiacus* MCR can be split into two functional fragments, MCR-N and MCR-C presenting alcohol dehydrogenase and aldehyde dehydrogenase (CoA-acylating) activities, respectively. Separating MCR into MCR-N and MCR-C fragments resulted in increased enzyme activity and 3HP production in a recombinant *E. coli* strain. Differently with constructing fusion proteins to improve metabolic efficiency and product yield, this study showed feasibility to develop a new strategy for metabolic engineering.
